# Effective management of atherosclerosis progress and hyperlipidemia with nattokinase: A clinical study with 1,062 participants

**DOI:** 10.3389/fcvm.2022.964977

**Published:** 2022-08-22

**Authors:** Hongjie Chen, Jiepeng Chen, Fuping Zhang, Yuanhui Li, Ronghua Wang, Qiang Zheng, Xu Zhang, Jun Zeng, Feng Xu, Yiguang Lin

**Affiliations:** ^1^Department of Traditional Chinese Medicine, Third Affiliated Hospital of Sun Yat-sen University, Guangzhou, China; ^2^Sungen Bioscience Co. Ltd., Shantou, China; ^3^Department of Pharmacy, Shenyang Red Cross Hospital, Shenyang, China; ^4^Guangzhou Center, Sinopharm Group Pharmaceutical Co., Ltd., Guangzhou, China; ^5^Antithrombotic & Thrombolytic Innovative Drug Research Center, Shenyang Pharmaceutical University, Shenyang, China; ^6^The Central Laboratory, First Affiliated Hospital of Guangdong Pharmaceutical University, Guangzhou, China; ^7^School of Life Sciences, University of Technology Sydney, Sydney, NSW, Australia

**Keywords:** nattokinase, atherosclerosis, hyperlipidaemia, anti-atherogenic drug, lipid lowering effect, retrospective study, lipid lowering agent, Nattokinase (NK)

## Abstract

Nattokinase (NK), known as a potent fibrinolytic and antithrombotic agent, has been shown to have antiatherosclerotic and lipid-lowering effects. However, data on human clinical studies are limited. In this clinical study involving 1,062 participants, our objective was to examine the efficacy of NK in atherosclerosis and hyperlipidemia and safety at the dose of 10,800 FU/day after 12 months of oral administration. Various factors, including lower doses that influence NK pharmacological actions, were also investigated. We found that NK at a dose of 10,800 FU/day effectively managed the progression of atherosclerosis and hyperlipidemia with a significant improvement in the lipid profile. A significant reduction in the thickness of the carotid artery intima-media and the size of the carotid plaque was observed. The improvement rates ranged from 66.5 to 95.4%. NK was found to be ineffective in lowering lipids and suppressing atherosclerosis progression at a dose of 3,600 FU/day. The lipid-lowering effect of NK was more prominent in subjects who smoked, drank alcohol, and subjects with higher BMI. Regular exercise further improved the effects of NK. Co-administration of vitamin K2 and aspirin with NK produced a synergetic effect. No noticeable adverse effects associated with the use of NK were recorded. In conclusion, our data demonstrate that atherosclerosis progression and hyperlipidemia can be effectively managed with NK at a dose of 10,800 FU/day. The lower dose of 3,600 FU per day is ineffective. The dose of 10,800 FU/day is safe and well tolerated. Some lifestyle factors and the coadministration of vitamin K2 and aspirin lead to improved outcomes in the use of NK. Our findings provide clinical evidence on the effective dose of NK in the management of cardiovascular disease and challenge the recommended dose of 2,000 FU per day.

## Introduction

Nattokinase (NK), the most active ingredient in natto with an alkaline protease of 275 amino acid residues, molecular weight approximately 28 kDa, was first discovered by Sumi et al. in 1987 (1). It is a potent fibrinolytic enzyme (2) demonstrating many favorable effects on cardiovascular health (3). The effects of NK include antihypertensive, anti-atherosclerotic, lipid lowering, anti-platelet and neuroprotective effects (3). Natto consumption is believed to be a significant contributor to the longevity of the Japanese population and a high intake of natto is associated with a decreased risk of total cardiovascular disease mortality (4). NK as a health supplement has been distributed throughout the world and has gained popularity among people who want to actively prevent cardiovascular disease.

Previous studies have demonstrated that NK and NK-containing natto have anti-atherosclerotic and lipid lowering effects (3, 5). Dietary natto extract supplementation suppresses intimal thickening in rats when compared to the control group (6, 7). The suppression of intimal thickening after vascular injury may be attributed to the enhanced thrombolytic activities of NK (6, 7). Chang et al. proposed that the natto extract suppressed intimal thickening through a synergistic effect attributed to its antioxidant and anti-apoptotic properties (8). Another study demonstrated that NK prevented arteriosclerosis by direct antioxidation leading to reduced lipid peroxidation and improved lipid metabolism (inhibition of LDL oxidation) (9). When used in combination with red ginseng, NK was found to reduce the area of aortic plaque in rabbits fed a hypercholesterol diet (10). We previously demonstrated that daily NK supplementation was an effective way to suppress the progression of atherosclerosis in patients with atherosclerotic plaques (5). In addition to its anti-atherosclerotic effects, NK or natto extract also has a favorable effect on lipids. Using NK or natto extract containing NK, animal studies from various laboratories confirmed that NK has a hypolipidemic effect and significantly reduces elevated serum triglycerides (TG), total cholesterol (TC) and low-density lipoprotein cholesterol levels (LDL-C) (10–16). Our studies found that in patients with hyperlipidaemia, NK treatment (26 weeks at 6,000 FU) reduced TC, LDL-C and TG, and increased the level of high-density lipoprotein cholesterol (HDL-C) (5).

However, data from human studies have not been consistent or conclusive. For example, in a small pilot study, Wu and colleagues observed a decrease in serum cholesterol, LDL-C and HDL-C in the NK treatment group following 8 weeks of treatment at a dose of 4,000 FU, although the difference was not statistically significant (17). In a recent report aimed at determining the effect of NK on the progression of subclinical atherosclerosis, it was concluded that NK supplementation at the dose of 2,000 FU does not have an effect on the progression of subclinical atherosclerosis in healthy individuals at low CVD risk (18). In contrast, in studies using higher doses of 6,000 FU (5) and 7,000 FU (19), NK was effective in lowering the level of total cholesterol, triglycerides and low-density lipoprotein cholesterol in hyperlipidemic patients, and was also effective in reducing the thickness of the median of the common carotid artery (CCA-IMT) and the size of the carotid plaque. Therefore, more studies are needed to determine the clinical effects of NK.

In the present study, we retrospectively analyzed data from 1,062 participants who received NK orally for 12 months to examine the safety and efficacy of NK in the treatment of atherosclerosis progression and hyperlipidemia. Multiple factors that may influence the effect of NK were also explored. This is the largest study to date designed to evaluate the clinical effects of NK in human subjects and to advance our understanding of the clinical potential of NK. We also provide novel insights into the optimal dose required for the most beneficial effects.

## Materials and methods

### Study design and participants

**Figure 1:**
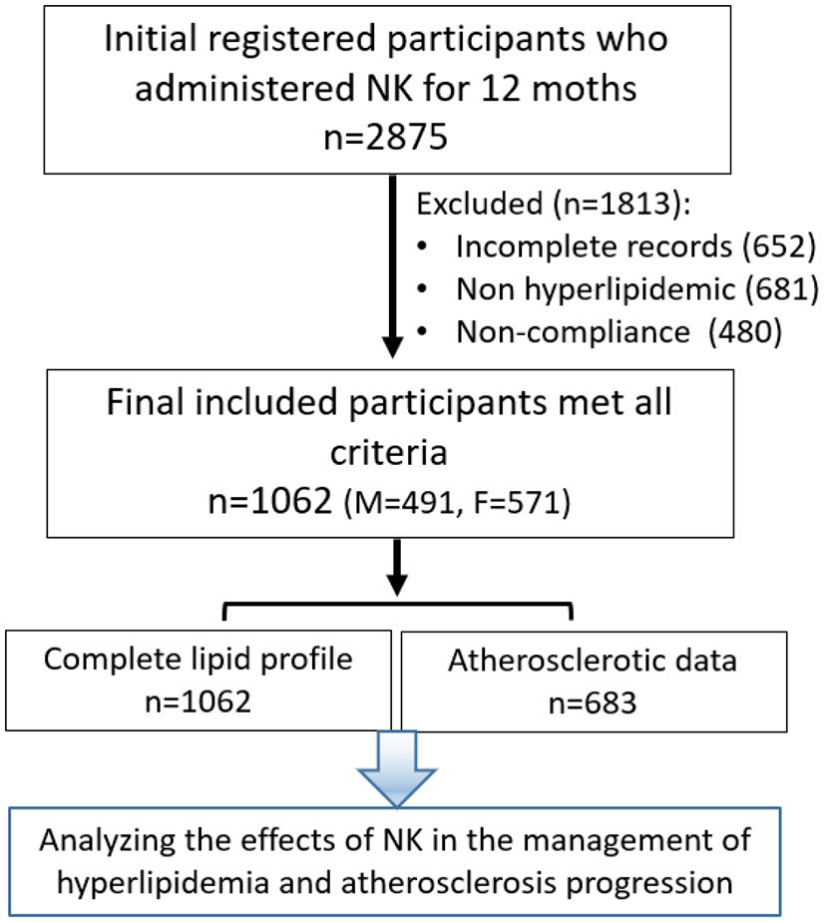
Flow Chat Illustrating the Design and Methods of the Study.

An initial total of 2,875 participant records were screened. Participants were selected from the Outpatient Clinic of the Third Affiliated Hospital of Sun Yat-sen University and Shenyang Red Cross Hospital, and 9 other Community Health Service Centers in Guangdong and Yunnan Provinces in China between January 2016 and June 2020. The participants included subjects who had marginal or mild hyperlipidemia and/or evidence indicating mild atherosclerosis and individuals who were particularly conscious about their health with a lipid profile within the normal range with some readings near the high end. Participants were recommended to take NK as an alternative health treatment in an attempt to improve their cardiovascular health conditions or who voluntarily took NK as a health supplement to improve/maintain cardiovascular health. Before and 12 months after NK use, all participants had blood lipid levels tested and ultrasound was performed to examine the common carotid artery for atherosclerotic evidence. The study design and methods are shown in the flow chart in Figure 1. After applying the criteria detailed below, 1,062 participants were included in the final study. This study was approved by the Human Ethics Committee of the Third Affiliated Hospital of Sun Yat-sen University (Approval number: 2015-2-92).

### Inclusion criteria and data collection

The inclusion criteria of the participants were: (1) evidence of hyperlipidemia; (2) available laboratory tests and ultrasound reports on the carotid artery before and 12 months after NK was used; (3) complete history of NK use at the required dose for 12 months. In data collection, two trained nurses checked all available records and excluded participants who had incomplete records, including baseline information, required laboratory tests, and ultrasound tests, did not comply with NK administration for 12 months, and were not hyperlipidemic. Two scientists evaluated the records and extracted data for retrospective analysis.

### Nattokinase administration

Nattokinase used throughout the study was administered orally as a nattokinase tablet approved by the China National Medical Products Administration (NMPA) and manufactured by Sungen Bioscience Co, Ltd, Shantou, China. Each tablet contained 3,600 FU. The oral dosage used was 10,800 FU daily. Compliance was monitored weekly by the community center's health care staff. In a small number of participants, vitamin K2 (Sungen Bioscience Co, Ltd, Shantou) was co-administered at a dose of 180 μg daily. Some participants were co-administered aspirin (100 mg daily).

### Laboratory and ultrasound tests

#### Evidence of atherosclerosis

The Phillips IE33 color Doppler ultrasound diagnostic instrument with a probe frequency of 7.5 MHz was used to detect the participant's bilateral common carotid arteries and two-dimensional ultrasound to observe the wall, plaque and lumen of the common carotid artery. The stenosis rate and CCA-IMT were measured in the thickest part of the IMT on the long axis of the carotid artery. The size of the plaque was measured according to the method of Spence et al. (20).

#### Hyperlipidaemia

Fasting venous blood was collected from each participant before treatment and 12 months after treatment. The measurement taken included the level of total cholesterol, triacylglycerol, high-density lipoprotein and low-density lipoprotein. Liver and kidney function were also tested.

### Factors that may influence the effects of NK

The effect of NK can be influenced by factors such as gender, NK dosage, participant lifestyle, body mass index, smoking status, alcohol consumption, coadministration of other agents such aspirin and vitamin K2. In the current study, information related to these factors was used to examine whether they influenced the effect of NK.

When considering the amount of exercise, step counting is used as an index to categorize participants as sedentary or not. As described in previous studies (21), 5,000 steps per day were used as the cut-off point to classify participants into two groups: <5,000 steps / day (sedentary / non-exercise group) and >5,000 steps / day (non-sedentary / exercise group).

For most of the participants, the dose used in the study was 10,800 FU daily. A small dose of 3,600 FU per day was used in one group for comparison.

BMI was calculated using body weight in kilograms divided by the square of height in meters. The BMI was classified into two groups as non-obese (BMI < 27.4 kg/m^2^) and obese (BMI ≥ 27.5 kg/m^2^) according to the obesity criteria of the World Health Organization (WHO) for Asians (22).

Alcohol consumption was divided into two categories; consumption of alcohol over 100 g per week on average was categorized in the alcohol group and below 100 g per week was categorized in the non-alcohol group. The use of a threshold of 100 g/week is based on a previous study by Wood et al. (23).

The smoking group referred to subjects who smoked on a daily basis regardless of the number of cigarettes used per day and compared to non-smokers.

Some participants used vitamin K2 (180 μg/day) regularly in addition to NK. Therefore, we collected data to analyze whether vitamin K2 influenced the action of NK.

The use of a low dose of aspirin was relatively common. Therefore, we also analyzed whether co-administration of low doses of aspirin and NK resulted in a different clinical outcome.

### Statistical analysis

Statistical analysis was carried out with SPSS (version 26, USA). Data were presented as mean ± SD. The paired t test is used for the comparison of each group before and after treatment, and the q test is used for the pairwise comparison between the groups. Statistical significance was set at *P* < 0.05.

## Results

### Profile of participants

A total of 1062 participants who used NK for 12 months were included in the study. The cohort participants included 491 men and 571 women, with an average age of 67.5 years and an age distribution of 63–85 years. The general and baseline information of the participants is summarized in Table 1.

**Table 1:** Baseline characteristics of the participants.

Age (year)	67.5 (63–85)
**Gender**
Male	491
Female	571
**Exercise**
<5,000 steps/d	356
>5,000 steps/d	706
**BMI**
<27.5	980
>27.5	82
Smoker	259
**Alcohol drinking**
<100 ml/w	799
>100 ml/w	263
Vitamin K2 co-admin (180 μg/d)	181
Aspirin co-admin (100 mg/d)	96
Hyperlipidemia	1,062
Atherosclerosis	683

### Effects of NK use on the lipid profile

The changes in the blood lipid profile of the participants before and after treatment are shown in Table 2. After 12 months of daily NK consumption at a dose of 10,800 FU, a significant reduction in TG, TC, and LDL-C (*P* < 0.01) was evident compared to the values before treatment. Furthermore, NK also had the effect of increasing HDL-C (15.8% increase, *P* < 0.01). The levels of TC, TG, LDL-C, and HDL-C improved in 95.4, 85.2, 84.3, and 89.1% of the participants, respectively, after 12 months of NK use (Table 3). NK administration for 12 months led to a decrease of 15.9, 15.3, and 18.1% in TC, TG, and LDL-C, respectively. Taking all of the data into account, NK produced a significant and favorable effect on the lipid profile in hyperlipidemic participants.

**Table 2:** Effect of daily NK consumption (10,800 FU daily) for 12 months on the lipid profile.

**Item**	**Sample Size**	**Before**	**After**	**Change %**
TG (mmol/L)	1,062	1.85 ± 0.65	1.56 ± 0.63^**^	−15.7
TC (mmol/L)	1,062	5.65 ± 0.96	4.75 ± 0.85^**^	−15.9
LDL-C (mmol/L)	1,062	3.58 ± 0.71	2.93 ± 0.67^**^	−18.1
HDL-C (mmol/L)	1,062	1.38 ± 0.56	1.60 ± 0.35^**^	15.8

*Compared the value after treatment with that before treatment*

** *p < 0.01*.

**Table 3:** Overall improvement rates following 12 months NK consumption (10,800 FU daily).

**Item**	**Total Case**	**Improved Case**	**Unimproved case**	**Improvement rate %**
TC	1,062	1,013	49	95.4
TG	1,062	905	157	85.2
LDL-C	1,062	895	167	84.3
HDL-C	1,062	946	116	89.1
CCA-IMT	683	531	152	77.7
CPS	683	454	229	66.5

*CPS, size of the carotid plaque, CCA-IMT, thickness of the intima-media of the common carotid artery*.

### NK suppresses atherosclerosis

After 12 months of NK consumption, both the size of CCA-IMT and the size of the carotid artery plaque decreased significantly (from 1.33 to 1.04mm on average, *P* < 0.001). The size of the plaque decreased by up to 36%, suggesting that NK is very effective in improving/reducing carotid atherosclerosis (Table 4). The overall improvement rates in CCA-IMT and CPS are not as high as those in blood lipids, with approximately 2/3 and 77.7% of the participants showing improvement in CPS and CCA-IMT, respectively (Table 3).

**Table 4:** Changes in the thickness of the intima-media of the common carotid artery (CCA-IMT, mm) and the size of the carotid plaque (CPS, mm^2^) after 12 months of daily NK consumption.

**Item**	**Sample Size**	**Before**	**After**	**Change %**
CCA-IMT (mm)	683	1.33 ± 0.81	1.04 ± 0.56^**^	−21.7
CPS (mm^2^)	683	24.9 ± 9.8	15.94 ± 7.3^**^	−36.0

*Compared to the value before treatment*,

** *p < 0.01. NK dose = 10,800 FU daily*.

### Other factors contributing to NK's actions

As shown in Table 5, no differences in NK efficacy were found between male and female participants, although changes in female participants were marginally greater, but were not statistically significant. We found that NK at 3,600 FU dose was not effective in lowering lipids and suppressing atherosclerosis. Lipid levels and CCA-IMT and plaque size did not change after 12 months of NK consumption at that dose (Table 6).

**Table 5:** Comparison of the effects of NK on lipid profile and atherosclerosis progression in male and female participants.

**Items**	**Male (*****n*** = **491)**			**Female (*****n*** = **571)**		
	**Before**	**After**	**Change%**	**Before**	**After**	**Change%**
TG (mmol/L)	2.10 ± 0.86	1.78 ± 0.61	−15.2	1.76 ± 0.95	1.47 ± 0.69	−16.6
TC (mmol/L)	5.92 ± 0.59	5.0 ± 0.77	−15.5	5.51 ± 0.66	4.60 ± 0.80	−16.5
LDL-C (mmol/L)	3.64 ± 0.67	3.02 ± 0.69	−17.1	3.46 ± 0.65	2.84 ± 2.32	−18.1
HDL-C (mmol/L)	1.25 ± 0.23	1.44 ± 0.39	14.9	1.39 ± 0.23	1.63 ± 0.57	17.0
CCA-IMT (mm)	1.36 ± 0.72	1.09 ± 0.67	−19.0	1.18 ± 0.67	0.98 ± 0.71	−22.9
CPS (mm^2^)	25.02 ± 13.27	16.16 ± 9.59	−35.4	24.35 ± 11.43	15.46 ± 12.86	−36.6

**Table 6:** Comparison of the effects of NK at two different doses on the lipid profile and atherosclerosis progression.

**Item**	**3,600 FU (*****n*** = **61)**			**10800 FU (*****n*** = **1,001)**		
	**Before**	**After**	**Change%**	**Before**	**After**	**Change%**
TG (mmol/L)	1.99 ± 0.88	1.97 ± 0.62	−1.0	1.73 ± 0.98	1.46 ± 0.78^**^	−15.5
TC (mmol/L)	5.82 ± 0.56	5.60 ± 0.72	−3.8	5.59 ± 0.93	4.68 ± 0.65^**^	−16.3
LDL-C (mmol/L)	3.54 ± 0.68	3.62 ± 0.69	2.3	3.64 ± 0.65	3.02 ± 1.02^**^	−17.1
HDL-C (mmol/L)	1.29 ± 0.33	1.33 ± 0.23	3.1	1.35 ± 0.53	1.57 ± 0.32^**^	16.1
CCA-IMT (mm)	1.25 ± 0.65	1.20 ± 0.34	−4.0	1.36 ± 0.71	1.06 ± 0.54^**^	−22.1
CPS (mm^2^)	26.05 ± 12.7	25.12 ± 6.57	−3.5	24.61 ± 12.43	15.69 ± 11.60^**^	−36.2

*Compared to the value before treatment*,

** *p < 0.01*.

Table 7 showed that the lipid lowering and antiatherosclerotic effects of NK were better in participants who exercise more compared to those who exercised less. Compared the effects of NK in obese participants with those of non-obese subjects, we found that the effects of NK in obese subjects were more prominent (Table 8).

**Table 7:** Comparison of the effects of NK on lipid profile and progression of atherosclerosis in sedentary and non- sedentary participants.

**Item**	<**5,000 steps/day (*****n*** = **356)**			>**5,000 steps/day (*****n*** = **706)**		
	**Before**	**After**	**Change%**	**Before**	**After**	**Change%**
TG (mmol/L)	1.69 ± 0.83	1.43 ± 0.35	−15.6	1.87 ± 1.08	1.57 ± 0.67	−16.0
TC (mmol/L)	5.81 ± 0.73	4.88 ± 0.73	−16.0	5.56 ± 0.91	4.63 ± 0.66	−16.7
LDL-C (mmol/L)	3.62 ± 0.76	2.97 ± 0.86	−17.9	3.53 ± 0.58	2.89 ± 0.65	−18.2
HDL-C (mmol/L)	1.31 ± 0.53	1.51 ± 0.36	14.9	1.37 ± 0.51	1.59 ± 0.52	16.2
CCA-IMT (mm)	1.28 ± 0.71	1.20 ± 0.34	−18.0	1.35 ± 0.61	1.05 ± 0.51	−22.0
CPS (mm^2^)	24.20 ± 13.2	16.70 ± 8.47	−31.0	25.50 ± 12.33	15.94 ± 10.02	−37.5

**Table 8:** Comparison of the effects of NK on lipid profile and progression of atherosclerosis progression in obese (BMI>27.5) and nonobese participants.

**Item**	**BMI**<**27.5 (*****n*** = **980)**			**BMI**>**27.5 (*****n*** = **82)**		
	**Before**	**After**	**Change%**	**Before**	**After**	**Change%**
TG (mmol/L)	1.73 ± 0.58	1.46 ± 0.65	−15.6	2.23 ± 0.93	1.86 ± 0.65	−16.6
TC (mmol/L)	5.59 ± 0.89	4.69 ± 0.72	−16.1	6.35 ± 0.86	5.24 ± 0.81	−17.5
LDL-C (mmol/L)	3.56 ± 0.61	2.94 ± 0.68	−17.5	3.93 ± 1.59	3.21 ± 1.32	−18.2
HDL-C (mmol/L)	1.35 ± 0.52	1.55 ± 0.39	15.1	1.25 ± 0.23	1.44 ± 0.38	15.3
CCA-IMT (mm)	1.31 ± 0.70	1.05 ± 0.57	−20.0	1.51 ± 0.65	1.16 ± 0.67	−23.1
CPS (mm^2^)	24.35 ± 13.27	15.73 ± 12.59	−35.4	32.2 ± 17.43	19.64 ± 17.86	−39.1

In smokers and alcohol-drinking participants, we found that hyperlipidemic conditions and atherosclerotic conditions were generally worse compared to nonsmokers and participants who drank less. We also found that the effects of NK on lipid lowering and antiatherosclerotic action were slightly stronger (Tables 9, 10).

**Table 9:** Comparison of the effects of NK on lipid profile and atherosclerosis progression in smoking and nonsmoking participants.

**Item**	**Smoker (*****n*** = **259)**			**Non-smoker (*****n*** = **803)**		
	**Before**	**After**	**Change%**	**Before**	**After**	**Change%**
TG (mmol/L)	2.11 ± 0.96	1.76 ± 0.76	−16.5	1.69 ± 0.78	1.42 ± 0.61	−16.0
TC (mmol/L)	6.28 ± 0.99	5.26 ± 0.79	−16.2	5.60 ± 0.78	4.73 ± 0.62	−15.6
LDL-C (mmol/L)	3.65 ± 0.67	3.03 ± 0.89	−16.9	3.55 ± 0.77	2.94 ± 0.58	−17.1
HDL-C (mmol/L)	1.16 ± 0.32	1.36 ± 0.38	17.1	1.43 ± 0.66	1.67 ± 0.59	16.5
CCA-IMT (mm)	1.39 ± 0.67	1.08 ± 0.56	−22.5	1.21 ± 0.54	0.97 ± 0.55	−20.2
CPS (mm^2^)	26.02 ± 13.12	16.25 ± 10.59	−37.5	24.71 ± 13.23	16.31 ± 11.69	−34.0

**Table 10:** Comparison of the effects of NK on the lipid profile and progression of atherosclerosis in alcohol and non-alcohol drinkers.

**Item**	**Alcohol intake**<**100 ml/w (*****n*** = **899)**			**Alcohol intake**>**100 ml/w (*****n*** = **163)**		
	**Before**	**After**	**Change%**	**Before**	**After**	**Change%**
TG (mmol/L)	1.63 ± 0.77	1.38 ± 0.56	−15.6	2.36 ± 0.96	1.97 ± 0.56	−16.6
TC (mmol/L)	5.62 ± 0.98	4.72 ± 0.56	−16.1	6.52 ± 1.66	5.43 ± 0.82	−16.7
LDL-C (mmol/L)	3.51 ± 0.68	2.91 ± 0.74	−17.0	4.02 ± 0.76	3.29 ± 1.32	−18.1
HDL-C (mmol/L)	1.38 ± 0.43	1.60 ± 0.59	16.3	1.32 ± 0.63	1.52 ± 0.67	15.0
CCA-IMT (mm)	1.27 ± 0.52	1.00 ± 0.56	−21.0	1.42 ± 0.63	1.09 ± 0.56	−23.0
CPS (mm^2^)	24.3 ± 12.71	15.80 ± 6.79	−35.0	35.01 ± 12.43	17.69 ± 11.72	−39.1

### Effect of coadministration of vitamin K2 and aspirin on the actions of NK

As shown in Table 11, co-administration of vitamin K2 and NK showed a synergistic effect on blood lipids in hyperlipidemic participants. There was no significant impact on other indicators of atherosclerosis. On the contrary, the simultaneous use of aspirin led to a synergistic effect on blood lipid profiles and suppressed atherosclerosis progression, meaning that favorable changes in the lipid profile and suppression of atherosclerosis progression were more significant (Table 12).

**Table 11:** Effect of co-administration of vitamin K2 (180 μg daily) on NK action.

**Item**	**With Vitamin K2 (*****n*** = **181)**			**Without Vitamin K2 (*****n*** = **881)**		
	**Before**	**After**	**Change%**	**Before**	**After**	**Change%**
TG (mmol/L)	1.93 ± 0.88	1.61 ± 0.64	−16.5	1.79 ± 0.77	1.51 ± 0.64	−15.6
TC (mmol/L)	5.66 ± 0.87	4.66 ± 0.55	−17.6	5.65 ± 0.97	4.73 ± 0.77	−16.3
LDL-C (mmol/L)	3.61 ± 0.68	2.96 ± 0.73	−17.9	3.52 ± 0.66	2.88 ± 0.91	−18.2
HDL-C (mmol/L)	1.42 ± 0.52	1.66 ± 0.46	17.0	1.37 ± 0.50	1.58 ± 0.53	15.5
CCA-IMT (mm)	1.31 ± 0.64	0.98 ± 0.64	−25.1	1.36 ± 0.74	1.07 ± 0.57	−21.1
CPS (mm^2^)	25.61 ± 13.74	15.62 ± 8.81	−39.1	24.81 ± 9.13	15.87 ± 12.30	−36.0

**Table 12:** Effect of coadministration of aspirin (100 mg daily) on NK actions.

**Items**	**With Aspirin (*****n*** = **96)**			**Without Aspirin (*****n*** = **966)**		
	**Before**	**After**	**Change%**	**Before**	**After**	**Change%**
TG (mmol/L)	2.11 ± 0.93	1.72 ± 0.33	−18.5	1.81 ± 0.67	1.53 ± 0.64	−15.5
TC (mmol/L)	6.22 ± 0.72	5.11 ± 0.84	−17.8	5.54 ± 0.57	4.65 ± 0.76	−16.0
LDL-C (mmol/L)	4.11 ± 0.86	3.36 ± 0.85	−18.0	3.53 ± 0.66	2.90 ± 0.91	−17.8
HDL-C (mmol/L)	1.31 ± 0.39	1.54 ± 0.23	17.2	1.38 ± 0.50	1.60 ± 0.53	16.0
CCA-IMT (mm)	1.21 ± 0.50	0.90 ± 0.51	−26.0	1.34 ± 0.72	1.05 ± 0.71	−21.5
CPS (mm^2^)	30.12 ± 13.66	18.21 ± 11.73	−39.5	24.51 ± 12.13	15.93 ± 12.30	−35.1

## Discussion

The present retrospective study was conducted to assess the efficacy of daily NK consumption in the treatment of hyperlipidemia and atherosclerosis in the Chinese population. In this study, we present evidence to show that continued NK supplementation at the dose of 10,800 FU daily for 12 months significantly decreased TC, TG, LDL-C and increased HDL-C in hyperlipidemic participants. Administration of NK effectively improved atherosclerotic conditions by significantly reducing CCA-IMT and CPS. The study also investigated the factors surrounding the use of NK and contributing to improving clinical outcomes. The findings of this study are very important, as they demonstrate that NK at a dose of 10,800 FU, a high dose compared to the recommended dose of 2,000 FU for use in Europe (24), is highly effective in the treatment of hyperlipidemia and progression of atherosclerosis, two main contributors to the development of CVD.

The underlying mechanisms by which NK lowers lipids and suppresses atherosclerosis are not fully understood. Early studies indicated that NK enhances thrombolytic activities that contributed to the antiatherosclerotic effect (6, 7). The available data suggest that the antiatherosclerotic effects of NK are due to the collective effects of the combination of the antithrombotic, anticoagulant, antioxidant and lipid lowering properties of NK or the natto extract containing NK (5, 9, 10, 12). For some time, NK has been known to have a favorable effect on lipids. Animal studies from various laboratories using NK, or natto extract containing NK, confirm that NK has a clear hypolipidemic effect and significantly reduces the increased serum levels of TG, TC and LDL-C (10–16). The lipid lowering properties of NK have been confirmed in various clinical studies (5, 17, 19). Understanding how NK reduces / changes the lipid profile is limited. A possible mechanism is through NK proteolytic activity on certain protein targets involved in lipid metabolism, resulting in changes in lipid metabolism (19). What is already known is that a high dose of NK is required to achieve hypolipidemic effects (3).

An important finding from this study is that NK, when used in a high dose, is very effective both in controlling the progression of atherosclerosis and in lowering blood lipids. This is entirely consistent with previous findings in human clinical studies in which NK was used at doses of 6,000 FU and 7,000 FU (5, 19). However, current findings are not consistent with the findings of studies using low doses of NK (18). Historically, information on the optimal proposed dose of NK has caused confusion. Although NK has been used worldwide, the doses used vary greatly between studies. In clinical studies completed to date, the daily dose between studies can vary up to 10 times. Daily doses range from as low as 1,200 FU (25); 2,000 FU (18, 25–30); 3,000 FU (31); 4,000 FU (17) to higher doses, 6,000 FU (5), 7,000 FU (19) and 13,000 FU (32). We believe that the reason for the wide discrepancies and inconsistencies in the doses is that there have been no authoritative data on the effective dose (ED) or effective concentration (EC) of NK, the minimum effective dose (MED) and the maximum effective dose or the maximum tolerated dose (MTD). For NK to be used as an effective and reliable treatment or supplement for health benefits, it is necessary to establish ED, EC, and MED for NK under defined clinical conditions. Given the finding of this study, previous studies (5, 19) and animal studies (11), we believe that the ED of NK for effective treatment of atherosclerosis and hyperlipidemia is in the range of 6,000 to 12,000 FU daily, which is much higher than 2,000 FU, a recommended dose in Europe (24).

The use of the dose of 10,800 FU daily is based on previous studies demonstrating that NK is very safe without concerns of toxicity. In addition to the long history of the use of natto and purified NK in the diet in Asian countries, especially Japan, it has been shown that there is no concern for toxicity when adults take 1,000–14,000 FU daily (32), and no toxic side effects have been observed in rats using significantly higher doses of 22,000 FU/kg/day, equivalent to 1.43 million FU daily in humans (32). Importantly, there are no cases of toxic effects or serious side effects reported using this high dose in the literature, even though NK has been widely used and studied over many years. However, a side effect-related report showed that a patient with mechanical valve developed thrombus, but underwent a successful repeat valve replacement when using NK (100 mg daily, equivalent to 2,000 FU/day) as a replacement for warfarin (33). Again, this thrombus development problem may be related to the inefficiency of the low-dose used.

In this study, we found that the action of NK on lipid lowering and suppression of atherosclerosis was more prominent in people who exercised more frequently and were obese. It can be interpreted that participants who regularly exercise have a more disciplined lifestyle with better compliance, which can be favorable for improving the lipid profile and atherosclerosis regression. As shown in Table 6, the baseline for participants with a BMI>27.5 was much higher with more room for improvement. A better outcome was also observed in participants who smoked and consumed alcohol. We believe that the interpretation of this outcome in subjects who smoked and consumed alcohol is similar to that observed in obese participants, implying that people in these categories would have greater health benefits using NK supplements in their diet. The observation that co-administration of vitamin K2 and aspirin with NK led to a synergistic effect is interesting. In support of our findings, previous studies found that NK and aspirin share similar pathways and mechanisms of action in their interaction with platelets leading to inhibition of platelet aggregation (16, 34). Furthermore, the positive *in vitro* hemorheological effects of NK worked well with aspirin (35). These shared actions might contribute to a better clinical outcome. It is unknown why the use of vitamin K2 improved the action of NK. It could be related to the positive effect on bone, muscle and cardiovascular health associated with the administration of vitamin K2 (36, 37).

There are some limitations associated with this study. First, although the sample size of the current study is reasonably large, it represents a retrospective study and not a randomized control trial. The availability and reliability of information from the current study is limited. For example, self-claiming information on lifestyle cannot be validated. Second, this study does not include follow-up interviews. Therefore, there is no current information on the long-term benefits of the participants, and the study is fully based on the records taken previously. Third, there are no further data available to allow us to investigate the underlying mechanism of NK. However, we believe that the limitations are outweighed by the notable strengths and important findings of the study.

## Conclusions

In summary, our data from this largest clinical study involving 1,062 participants suggest that NK at the daily dose of 10,800 FU, which is higher than the recommended dose of 2,000 FU, is significantly effective in the management of atherosclerosis progression and hyperlipidemia. No adverse effects associated with the use of NK is observed. The study advances our understanding of the action of NK and the importance of the dosage of NK. We also demonstrate that other factors, including lifestyle and co-use of vitamin K2 and aspirin, could contribute positively to the clinical outcome. Our findings provide evidence that promising and positive clinical outcome in the management of atherosclerosis progression and hyperlipidemia can be achieved safely by using NK at a dose of 10,800 FU per day. The outcome of this report warrants further randomized control clinical trials using increased doses of NK.

## Data availability statement

The data used to support the findings of this study are included within the article.

## Ethics statement

The studies involving human participants were reviewed and this study was approved by the Human Ethics Committee of the Third Affiliated Hospital of Sun Yat-sen University (Approval number: 2015-2-92). Written informed consent for participation was not required for this study in accordance with the national legislation and the institutional requirements.

## Author contributions

HC, JC, FX, and YLin: conceptualization. HC, JC, FZ, YLi, RW, QZ, XZ, and JZ: methodology. YLin: software. HC, JC, FZ, YLi, RW, QZ, XZ, JZ, and FX: validation, investigation, and data curation. FX: formal analysis. FX, FZ, and YLin: resources. HC, FX, and YLin: writing–original draft preparation and visualization. FX and YL: writing–review and editing and project administration. FX, JC, and YLin: supervision. All authors have read and agreed to the published version of the manuscript.

## Conflict of interest

Authors JC, RW, QZ, XZ, and JZ were employed by Sungen Bioscience Co. Ltd. Author YLi was employed by Sinopharm Group Pharmaceutical Co., Ltd. The remaining authors declare that the research was conducted in the absence of any commercial or financial relationships that could be construed as a potential conflict of interest.

## Publisher's note

All claims expressed in this article are solely those of the authors and do not necessarily represent those of their affiliated organizations, or those of the publisher, the editors and the reviewers. Any product that may be evaluated in this article, or claim that may be made by its manufacturer, is not guaranteed or endorsed by the publisher.
