# Corrigendum: Effective management of atherosclerosis progress and hyperlipidemia with nattokinase: A clinical study with 1,062 participants

**DOI:** 10.3389/fcvm.2022.1076420

**Published:** 2022-12-05

**Authors:** Hongjie Chen, Jiepeng Chen, Fuping Zhang, Yuanhui Li, Ronghua Wang, Qiang Zheng, Xu Zhang, Jun Zeng, Feng Xu, Yiguang Lin

**Affiliations:** ^1^Department of Traditional Chinese Medicine, Third Affiliated Hospital of Sun Yat-sen University, Guangzhou, China; ^2^Sungen Bioscience Co. Ltd., Shantou, China; ^3^Department of Pharmacy, Shenyang Red Cross Hospital, Shenyang, China; ^4^Guangzhou Center, Sinopharm Group Pharmaceutical Co., Ltd., Guangzhou, China; ^5^Antithrombotic & Thrombolytic Innovative Drug Research Center, Shenyang Pharmaceutical University, Shenyang, China; ^6^The Central Laboratory, First Affiliated Hospital of Guangdong Pharmaceutical University, Guangzhou, China; ^7^School of Life Sciences, University of Technology Sydney, Sydney, NSW, Australia

**Keywords:** nattokinase, atherosclerosis, hyperlipidaemia, anti-atherogenic drug, lipid lowering effect, retrospective study, lipid lowering agent, Nattokinase (NK)

In the published article, there was an error in Table 1 Baseline characteristics of the participants as published.

**Table 1:** Baseline characteristics of the participants.

Age (year)	67.5 (63–85)
**Gender**
Male	491
Female	571
**Exercise**
<5,000 steps/d	356
>5,000 steps/d	706
**BMI**
<27.5	980
>27.5	82
Smoker	259
**Alcohol drinking**
<100 ml/w	799
>100 ml/w	263
Vitamin K2 co-admin (180 μg/d)	181
Aspirin co-admin (100 mg/d)	96
Hyperlipidemia	1,062
Atherosclerosis	683

The corrected Table and correct format appear below.

The authors apologize for this error and state that this does not change the scientific conclusions of the article in any way. The original article has been updated.

